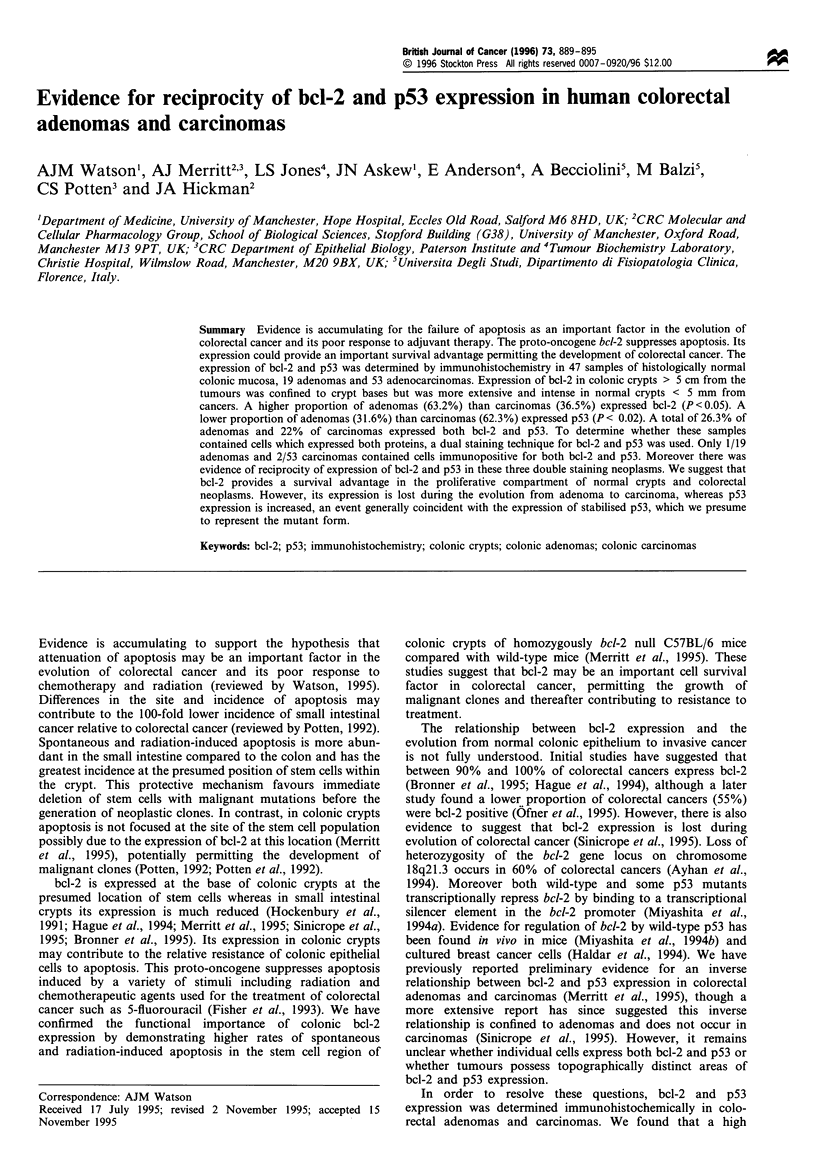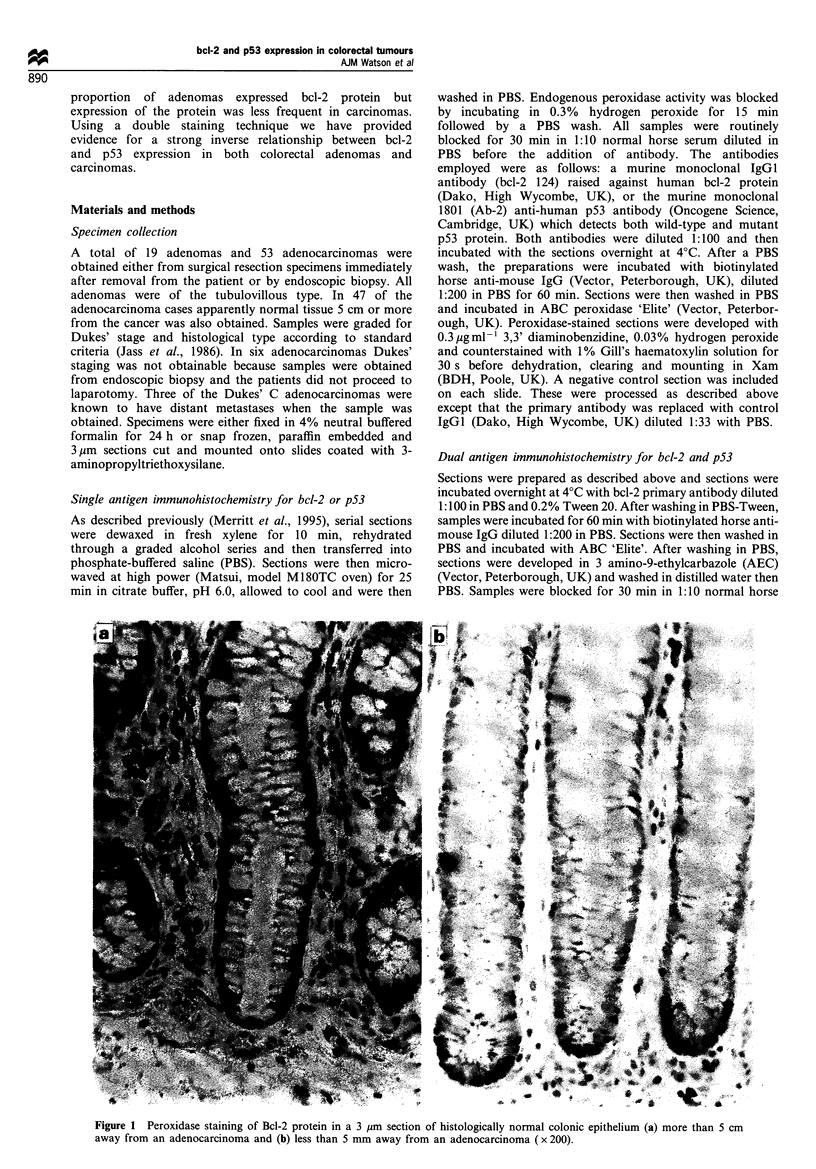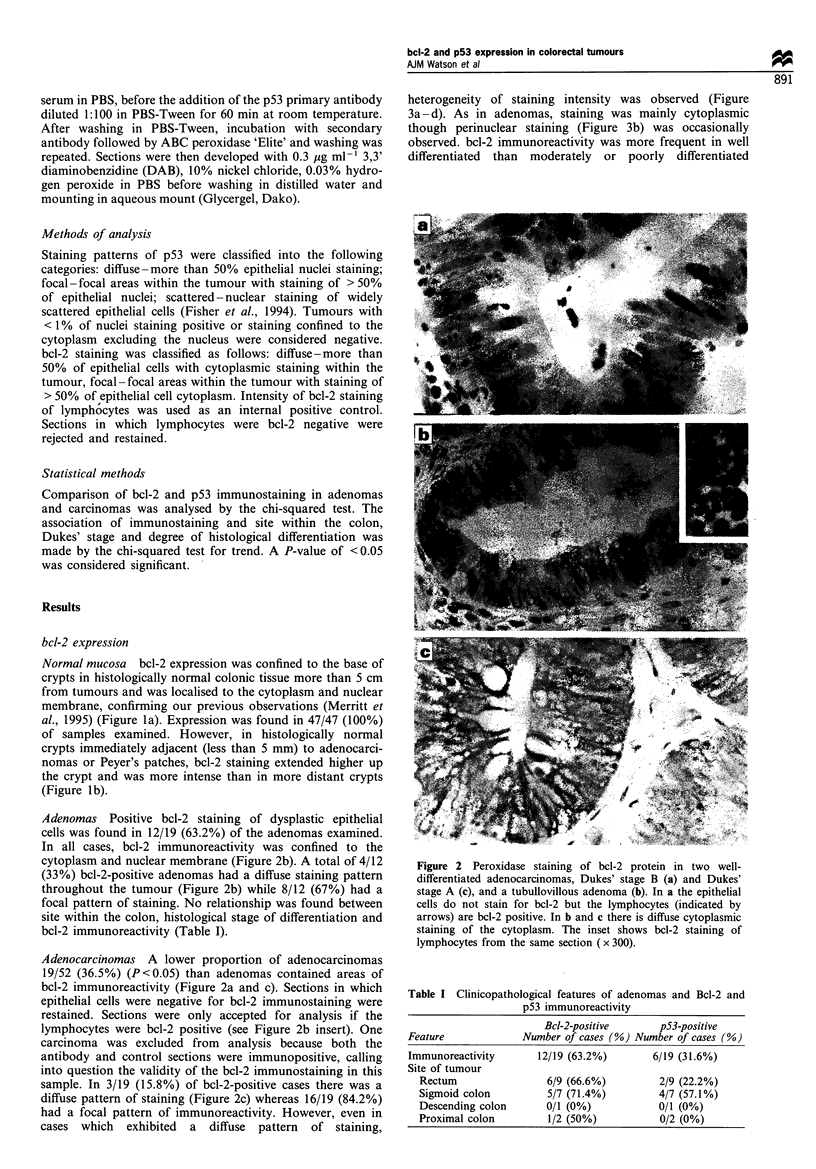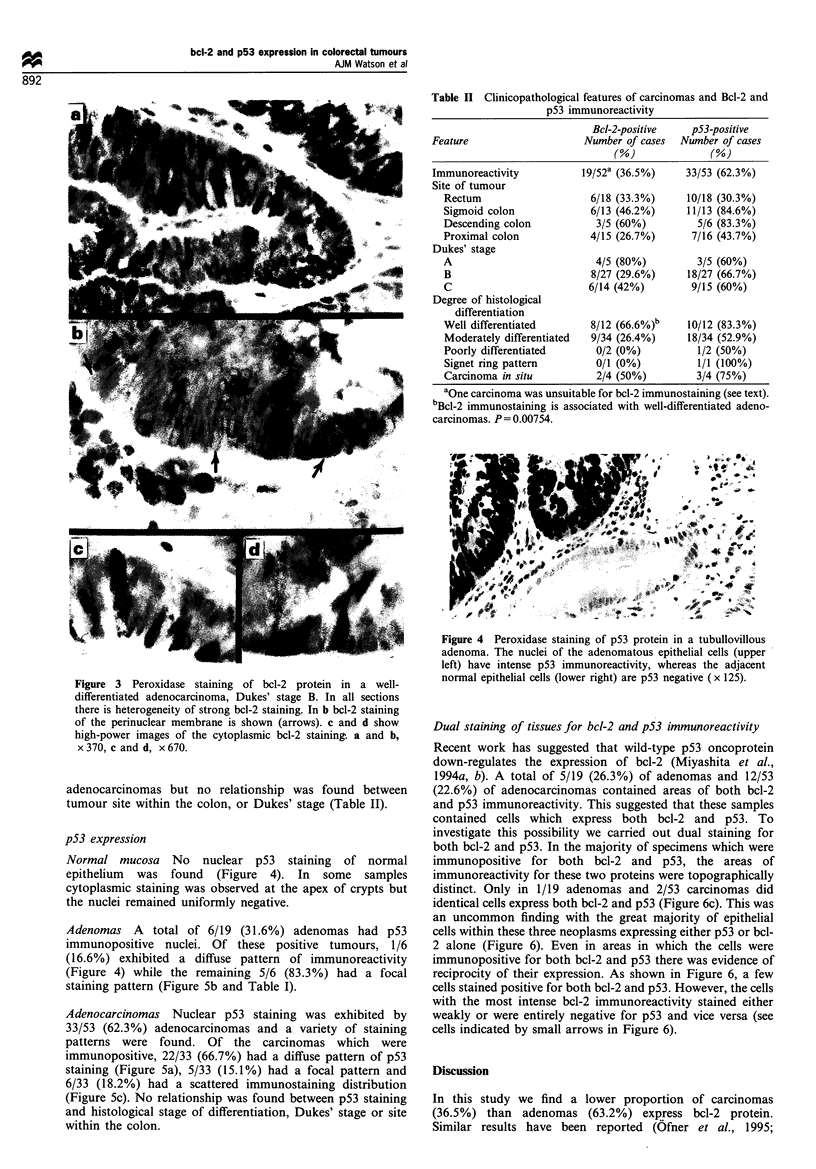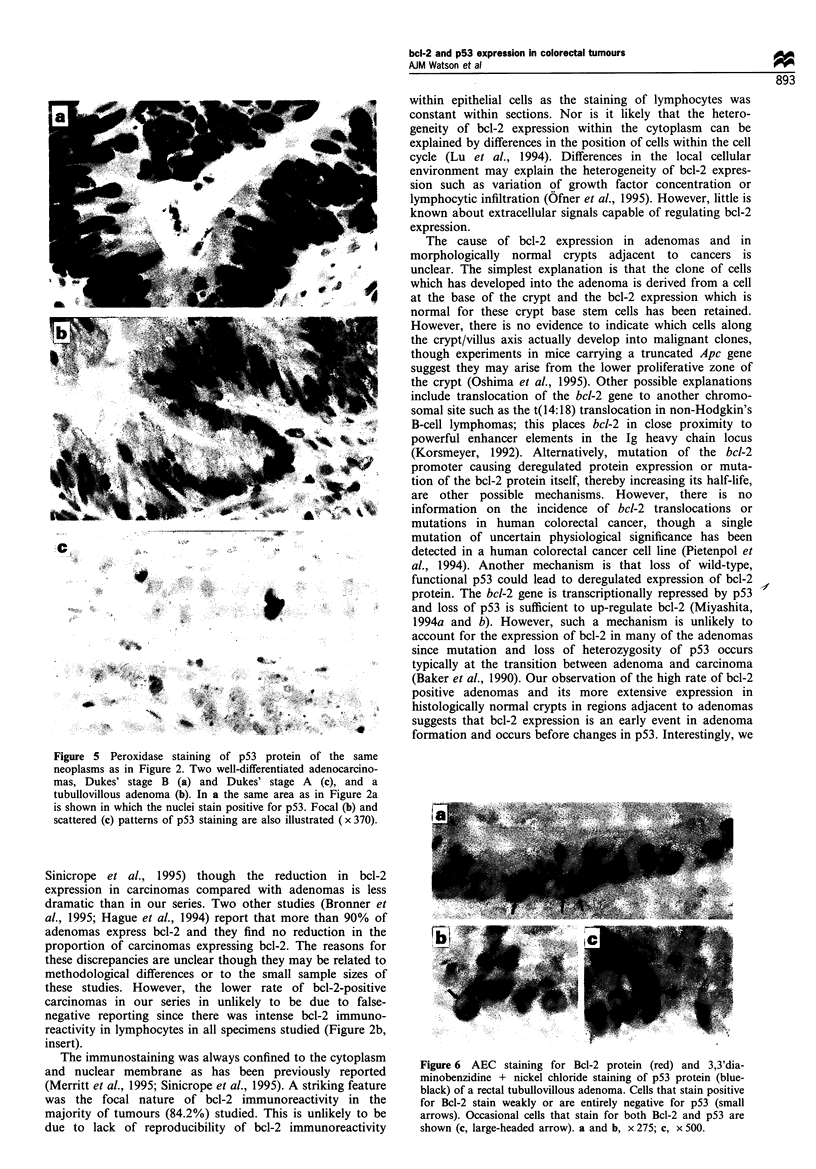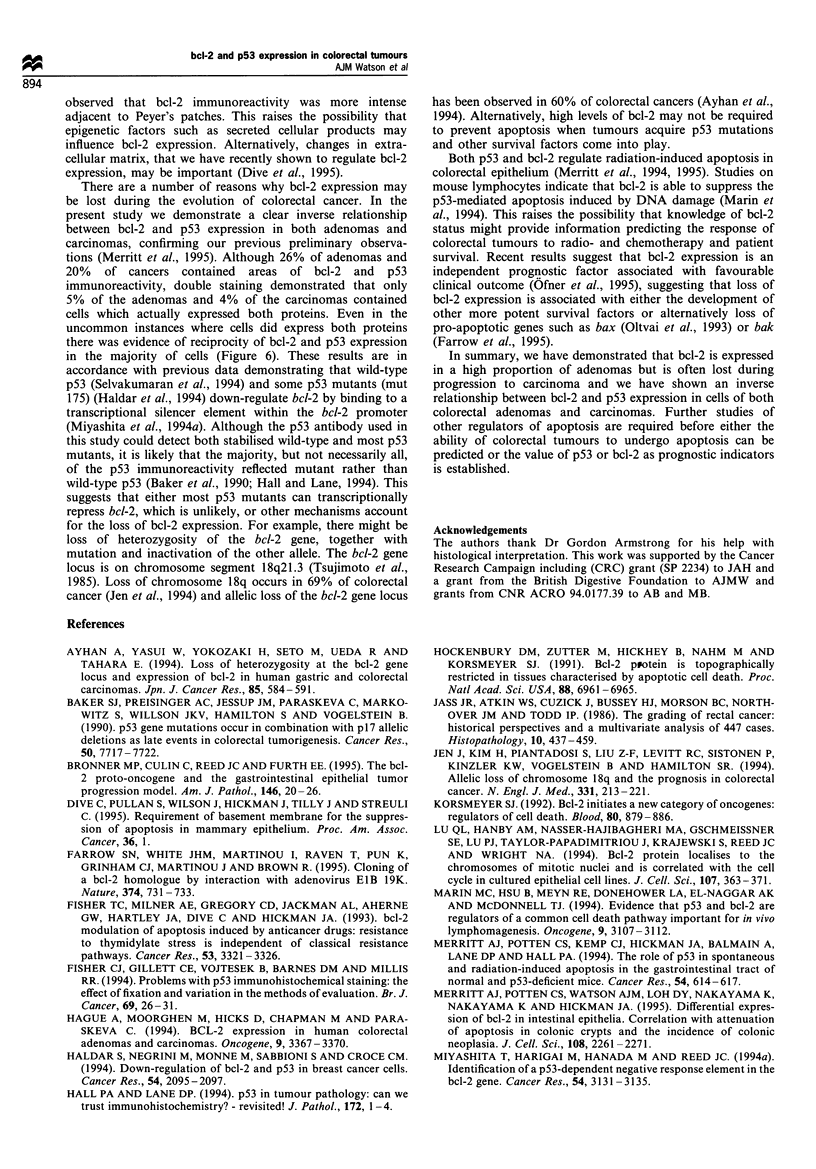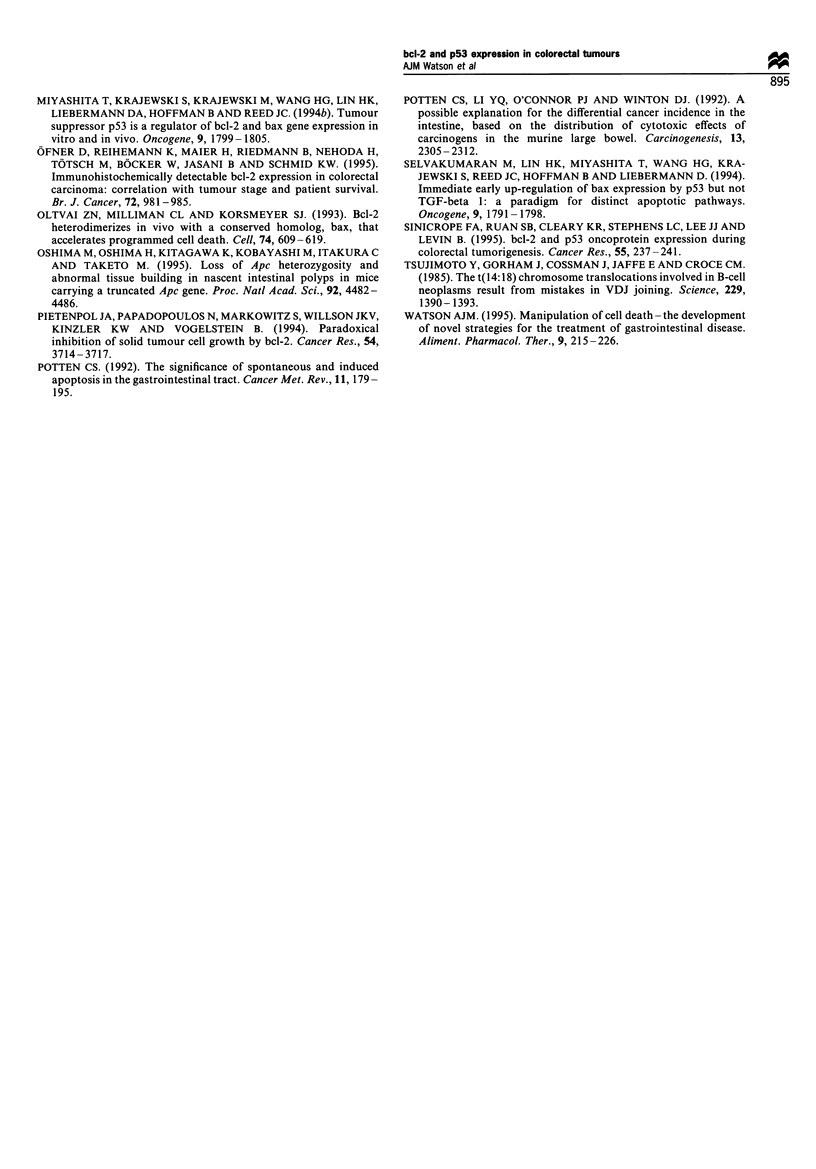# Evidence of reciprocity of bcl-2 and p53 expression in human colorectal adenomas and carcinomas.

**DOI:** 10.1038/bjc.1996.178

**Published:** 1996-04

**Authors:** A. J. Watson, A. J. Merritt, L. S. Jones, J. N. Askew, E. Anderson, A. Becciolini, M. Balzi, C. S. Potten, J. A. Hickman

**Affiliations:** Department of Medicine, University of Manchester, Hope Hospital, Salford, UK.

## Abstract

**Images:**


British Journal of Cancer (1996) 73, 889-895
© 1996 Stockton Press All rights reserved 0007-0920/96 $12.00

### Evidence for reciprocity of bcl-2 and p53 expression in human colorectal adenomas and carcinomas

AJM Watson[1], AJ Merritt[2,3], LS Jones[4], JN Askew[1], E Anderson[4], A Becciolini[5], M Balzi[5], CS Potten[3] and JA Hickman[2]

[1]*Department of Medicine, University of Manchester, Hope Hospital, Eccles Old Road, Salford M6 8HD, UK;* [2]*CRC Molecular and Cellular Pharmacology Group, School of Biological Sciences, Stopford Building (G38), University of Manchester, Oxford Road, Manchester M13 9PT, UK;* [3]*CRC Department of Epithelial Biology, Paterson Institute and* [4]*Tumour Biochemistry Laboratory, Christie Hospital, Wilmslow Road, Manchester, M20 9BX, UK;* [5]*Universita Degli Studi, Dipartimento di Fisiopatologia Clinica, Florence, Italy.*

**Summary** Evidence is accumulating for the failure of apoptosis as an important factor in the evolution of colorectal cancer and its poor response to adjuvant therapy. The proto-oncogene *bcl*-2 suppresses apoptosis. Its expression could provide an important survival advantage permitting the development of colorectal cancer. The expression of bcl-2 and p53 was determined by immunohistochemistry in 47 samples of histologically normal colonic mucosa, 19 adenomas and 53 adenocarcinomas. Expression of bcl-2 in colonic crypts > 5 cm from the tumours was confined to crypt bases but was more extensive and intense in normal crypts < 5 mm from cancers. A higher proportion of adenomas (63.2%) than carcinomas (36.5%) expressed bcl-2 ($P<0.05$). A lower proportion of adenomas (31.6%) than carcinomas (62.3%) expressed p53 ($P<0.02$). A total of 26.3% of adenomas and 22% of carcinomas expressed both bcl-2 and p53. To determine whether these samples contained cells which expressed both proteins, a dual staining technique for bcl-2 and p53 was used. Only 1/19 adenomas and 2/53 carcinomas contained cells immunopositive for both bcl-2 and p53. Moreover there was evidence of reciprocity of expression of bcl-2 and p53 in these three double staining neoplasms. We suggest that bcl-2 provides a survival advantage in the proliferative compartment of normal crypts and colorectal neoplasms. However, its expression is lost during the evolution from adenoma to carcinoma, whereas p53 expression is increased, an event generally coincident with the expression of stabilised p53, which we presume to represent the mutant form.

**Keywords:** bcl-2; p53; immunohistochemistry; colonic crypts; colonic adenomas; colonic carcinomas

Evidence is accumulating to support the hypothesis that attenuation of apoptosis may be an important factor in the evolution of colorectal cancer and its poor response to chemotherapy and radiation (reviewed by Watson, 1995). Differences in the site and incidence of apoptosis may contribute to the 100-fold lower incidence of small intestinal cancer relative to colorectal cancer (reviewed by Potten, 1992). Spontaneous and radiation-induced apoptosis is more abundant in the small intestine compared to the colon and has the greatest incidence at the presumed position of stem cells within the crypt. This protective mechanism favours immediate deletion of stem cells with malignant mutations before the generation of neoplastic clones. In contrast, in colonic crypts apoptosis is not focused at the site of the stem cell population possibly due to the expression of bcl-2 at this location (Merritt *et al.*, 1995), potentially permitting the development of malignant clones (Potten, 1992; Potten *et al.*, 1992).

bcl-2 is expressed at the base of colonic crypts at the presumed location of stem cells whereas in small intestinal crypts its expression is much reduced (Hockenbury *et al.*, 1991; Hague *et al.*, 1994; Merritt *et al.*, 1995; Sinicrope *et al.*, 1995; Bronner *et al.*, 1995). Its expression in colonic crypts may contribute to the relative resistance of colonic epithelial cells to apoptosis. This proto-oncogene suppresses apoptosis induced by a variety of stimuli including radiation and chemotherapeutic agents used for the treatment of colorectal cancer such as 5-fluorouracil (Fisher *et al.*, 1993). We have confirmed the functional importance of colonic bcl-2 expression by demonstrating higher rates of spontaneous and radiation-induced apoptosis in the stem cell region of colonic crypts of homozygously *bcl*-2 null C57BL/6 mice compared with wild-type mice (Merritt *et al.*, 1995). These studies suggest that bcl-2 may be an important cell survival factor in colorectal cancer, permitting the growth of malignant clones and thereafter contributing to resistance to treatment.

The relationship between bcl-2 expression and the evolution from normal colonic epithelium to invasive cancer is not fully understood. Initial studies have suggested that between 90% and 100% of colorectal cancers express bcl-2 (Bronner *et al.*, 1995; Hague *et al.*, 1994), although a later study found a lower proportion of colorectal cancers (55%) were bcl-2 positive (Öfner *et al.*, 1995). However, there is also evidence to suggest that bcl-2 expression is lost during evolution of colorectal cancer (Sinicrope *et al.*, 1995). Loss of heterozygosity of the *bcl*-2 gene locus on chromosome 18q21.3 occurs in 60% of colorectal cancers (Ayhan *et al.*, 1994). Moreover both wild-type and some p53 mutants transcriptionally repress *bcl*-2 by binding to a transcriptional silencer element in the *bcl*-2 promoter (Miyashita *et al.*, 1994*a*). Evidence for regulation of *bcl*-2 by wild-type p53 has been found *in vivo* in mice (Miyashita *et al.*, 1994*b*) and cultured breast cancer cells (Haldar *et al.*, 1994). We have previously reported preliminary evidence for an inverse relationship between bcl-2 and p53 expression in colorectal adenomas and carcinomas (Merritt *et al.*, 1995), though a more extensive report has since suggested this inverse relationship is confined to adenomas and does not occur in carcinomas (Sinicrope *et al.*, 1995). However, it remains unclear whether individual cells express both bcl-2 and p53 or whether tumours possess topographically distinct areas of bcl-2 and p53 expression.

In order to resolve these questions, bcl-2 and p53 expression was determined immunohistochemically in colorectal adenomas and carcinomas. We found that a high

Correspondence: AJM Watson
Received 17 July 1995; revised 2 November 1995; accepted 15 November 1995

proportion of adenomas expressed bcl-2 protein but expression of the protein was less frequent in carcinomas. Using a double staining technique we have provided evidence for a strong inverse relationship between bcl-2 and p53 expression in both colorectal adenomas and carcinomas.

### Materials and methods

### *Specimen collection*

A total of 19 adenomas and 53 adenocarcinomas were obtained either from surgical resection specimens immediately after removal from the patient or by endoscopic biopsy. All adenomas were of the tubulovillous type. In 47 of the adenocarcinoma cases apparently normal tissue 5 cm or more from the cancer was also obtained. Samples were graded for Dukes' stage and histological type according to standard criteria (Jass *et al.*, 1986). In six adenocarcinomas Dukes' staging was not obtainable because samples were obtained from endoscopic biopsy and the patients did not proceed to laparotomy. Three of the Dukes' C adenocarcinomas were known to have distant metastases when the sample was obtained. Specimens were either fixed in 4% neutral buffered formalin for 24 h or snap frozen, paraffin embedded and 3 $\mu$m sections cut and mounted onto slides coated with 3-aminopropyltriethoxysilane.

### *Single antigen immunohistochemistry for bcl-2 or p53*

As described previously (Merritt *et al.*, 1995), serial sections were dewaxed in fresh xylene for 10 min, rehydrated through a graded alcohol series and then transferred into phosphate-buffered saline (PBS). Sections were then microwaved at high power (Matsui, model M180TC oven) for 25 min in citrate buffer, pH 6.0, allowed to cool and were then washed in PBS. Endogenous peroxidase activity was blocked by incubating in 0.3% hydrogen peroxide for 15 min followed by a PBS wash. All samples were routinely blocked for 30 min in 1:10 normal horse serum diluted in PBS before the addition of antibody. The antibodies employed were as follows: a murine monoclonal IgG1 antibody (bcl-2 124) raised against human bcl-2 protein (Dako, High Wycombe, UK), or the murine monoclonal 1801 (Ab-2) anti-human p53 antibody (Oncogene Science, Cambridge, UK) which detects both wild-type and mutant p53 protein. Both antibodies were diluted 1:100 and then incubated with the sections overnight at 4°C. After a PBS wash, the preparations were incubated with biotinylated horse anti-mouse IgG (Vector, Peterborough, UK), diluted 1:200 in PBS for 60 min. Sections were then washed in PBS and incubated in ABC peroxidase 'Elite' (Vector, Peterborough, UK). Peroxidase-stained sections were developed with 0.3 $\mu g\, ml^{-1}$ 3,3' diaminobenzidine, 0.03% hydrogen peroxide and counterstained with 1% Gill's haematoxylin solution for 30 s before dehydration, clearing and mounting in Xam (BDH, Poole, UK). A negative control section was included on each slide. These were processed as described above except that the primary antibody was replaced with control IgG1 (Dako, High Wycombe, UK) diluted 1:33 with PBS.

### *Dual antigen immunohistochemistry for bcl-2 and p53*

Sections were prepared as described above and sections were incubated overnight at 4°C with bcl-2 primary antibody diluted 1:100 in PBS and 0.2% Tween 20. After washing in PBS-Tween, samples were incubated for 60 min with biotinylated horse anti-mouse IgG diluted 1:200 in PBS. Sections were then washed in PBS and incubated with ABC 'Elite'. After washing in PBS, sections were developed in 3 amino-9-ethylcarbazole (AEC) (Vector, Peterborough, UK) and washed in distilled water then PBS. Samples were blocked for 30 min in 1:10 normal horse

**Figure 1** Peroxidase staining of Bcl-2 protein in a 3 $\mu$m section of histologically normal colonic epithelium (**a**) more than 5 cm away from an adenocarcinoma and (**b**) less than 5 mm away from an adenocarcinoma ($\times$200).

serum in PBS, before the addition of the p53 primary antibody diluted 1:100 in PBS-Tween for 60 min at room temperature. After washing in PBS-Tween, incubation with secondary antibody followed by ABC peroxidase 'Elite' and washing was repeated. Sections were then developed with 0.3 $\mu$g ml$^{-1}$ 3,3' diaminobenzidine (DAB), 10% nickel chloride, 0.03% hydrogen peroxide in PBS before washing in distilled water and mounting in aqueous mount (Glycergel, Dako).

### *Methods of analysis*

Staining patterns of p53 were classified into the following categories: diffuse–more than 50% epithelial nuclei staining; focal–focal areas within the tumour with staining of >50% of epithelial nuclei; scattered–nuclear staining of widely scattered epithelial cells (Fisher *et al.*, 1994). Tumours with <1% of nuclei staining positive or staining confined to the cytoplasm excluding the nucleus were considered negative. bcl-2 staining was classified as follows: diffuse–more than 50% of epithelial cells with cytoplasmic staining within the tumour, focal–focal areas within the tumour with staining of >50% of epithelial cell cytoplasm. Intensity of bcl-2 staining of lymphocytes was used as an internal positive control. Sections in which lymphocytes were bcl-2 negative were rejected and restained.

### *Statistical methods*

Comparison of bcl-2 and p53 immunostaining in adenomas and carcinomas was analysed by the chi-squared test. The association of immunostaining and site within the colon, Dukes' stage and degree of histological differentiation was made by the chi-squared test for trend. A $P$-value of <0.05 was considered significant.

### Results

### *bcl-2 expression*

*Normal mucosa* bcl-2 expression was confined to the base of crypts in histologically normal colonic tissue more than 5 cm from tumours and was localised to the cytoplasm and nuclear membrane, confirming our previous observations (Merritt *et al.*, 1995) (Figure 1a). Expression was found in 47/47 (100%) of samples examined. However, in histologically normal crypts immediately adjacent (less than 5 mm) to adenocarcinomas or Peyer's patches, bcl-2 staining extended higher up the crypt and was more intense than in more distant crypts (Figure 1b).

*Adenomas* Positive bcl-2 staining of dysplastic epithelial cells was found in 12/19 (63.2%) of the adenomas examined. In all cases, bcl-2 immunoreactivity was confined to the cytoplasm and nuclear membrane (Figure 2b). A total of 4/12 (33%) bcl-2-positive adenomas had a diffuse staining pattern throughout the tumour (Figure 2b) while 8/12 (67%) had a focal pattern of staining. No relationship was found between site within the colon, histological stage of differentiation and bcl-2 immunoreactivity (Table I).

*Adenocarcinomas* A lower proportion of adenocarcinomas 19/52 (36.5%) ($P$<0.05) than adenomas contained areas of bcl-2 immunoreactivity (Figure 2a and c). Sections in which epithelial cells were negative for bcl-2 immunostaining were restained. Sections were only accepted for analysis if the lymphocytes were bcl-2 positive (see Figure 2b insert). One carcinoma was excluded from analysis because both the antibody and control sections were immunopositive, calling into question the validity of the bcl-2 immunostaining in this sample. In 3/19 (15.8%) of bcl-2-positive cases there was a diffuse pattern of staining (Figure 2c) whereas 16/19 (84.2%) had a focal pattern of immunoreactivity. However, even in cases which exhibited a diffuse pattern of staining, heterogeneity of staining intensity was observed (Figure 3a–d). As in adenomas, staining was mainly cytoplasmic though perinuclear staining (Figure 3b) was occasionally observed. bcl-2 immunoreactivity was more frequent in well differentiated than moderately or poorly differentiated

**Figure 2** Peroxidase staining of bcl-2 protein in two well-differentiated adenocarcinomas, Dukes' stage B (**a**) and Dukes' stage A (**c**), and a tubullovillous adenoma (**b**). In **a** the epithelial cells do not stain for bcl-2 but the lymphocytes (indicated by arrows) are bcl-2 positive. In **b** and **c** there is diffuse cytoplasmic staining of the cytoplasm. The inset shows bcl-2 staining of lymphocytes from the same section (×300).

**Table I** Clinicopathological features of adenomas and Bcl-2 and p53 immunoreactivity

| Feature | *Bcl-2-positive* *Number of cases (%)* | *p53-positive* *Number of cases (%)* |
|---|---|---|
| Immunoreactivity | 12/19 (63.2%) | 6/19 (31.6%) |
| Site of tumour | | |
| Rectum | 6/9 (66.6%) | 2/9 (22.2%) |
| Sigmoid colon | 5/7 (71.4%) | 4/7 (57.1%) |
| Descending colon | 0/1 (0%) | 0/1 (0%) |
| Proximal colon | 1/2 (50%) | 0/2 (0%) |

**Figure 3** Peroxidase staining of bcl-2 protein in a well-differentiated adenocarcinoma, Dukes' stage B. In all sections there is heterogeneity of strong bcl-2 staining. In **b** bcl-2 staining of the perinuclear membrane is shown (arrows). **c** and **d** show high-power images of the cytoplasmic bcl-2 staining. **a** and **b**, ×370, **c** and **d**, ×670.

**Table II** Clinicopathological features of carcinomas and Bcl-2 and p53 immunoreactivity

| *Feature* | *Bcl-2-positive Number of cases (%)* | *p53-positive Number of cases (%)* |
|---|---|---|
| Immunoreactivity | 19/52[a] (36.5%) | 33/53 (62.3%) |
| Site of tumour | | |
| Rectum | 6/18 (33.3%) | 10/18 (30.3%) |
| Sigmoid colon | 6/13 (46.2%) | 11/13 (84.6%) |
| Descending colon | 3/5 (60%) | 5/6 (83.3%) |
| Proximal colon | 4/15 (26.7%) | 7/16 (43.7%) |
| Dukes' stage | | |
| A | 4/5 (80%) | 3/5 (60%) |
| B | 8/27 (29.6%) | 18/27 (66.7%) |
| C | 6/14 (42%) | 9/15 (60%) |
| Degree of histological differentiation | | |
| Well differentiated | 8/12 (66.6%)[b] | 10/12 (83.3%) |
| Moderately differentiated | 9/34 (26.4%) | 18/34 (52.9%) |
| Poorly differentiated | 0/2 (0%) | 1/2 (50%) |
| Signet ring pattern | 0/1 (0%) | 1/1 (100%) |
| Carcinoma *in situ* | 2/4 (50%) | 3/4 (75%) |

[a]One carcinoma was unsuitable for bcl-2 immunostaining (see text). [b]Bcl-2 immunostaining is associated with well-differentiated adenocarcinomas. $P=0.00754$.

**Figure 4** Peroxidase staining of p53 protein in a tubullovillous adenoma. The nuclei of the adenomatous epithelial cells (upper left) have intense p53 immunoreactivity, whereas the adjacent normal epithelial cells (lower right) are p53 negative (×125).

adenocarcinomas but no relationship was found between tumour site within the colon, or Dukes' stage (Table II).

### *p53 expression*

*Normal mucosa* No nuclear p53 staining of normal epithelium was found (Figure 4). In some samples cytoplasmic staining was observed at the apex of crypts but the nuclei remained uniformly negative.

*Adenomas* A total of 6/19 (31.6%) adenomas had p53 immunopositive nuclei. Of these positive tumours, 1/6 (16.6%) exhibited a diffuse pattern of immunoreactivity (Figure 4) while the remaining 5/6 (83.3%) had a focal staining pattern (Figure 5b and Table I).

*Adenocarcinomas* Nuclear p53 staining was exhibited by 33/53 (62.3%) adenocarcinomas and a variety of staining patterns were found. Of the carcinomas which were immunopositive, 22/33 (66.7%) had a diffuse pattern of p53 staining (Figure 5a), 5/33 (15.1%) had a focal pattern and 6/33 (18.2%) had a scattered immunostaining distribution (Figure 5c). No relationship was found between p53 staining and histological stage of differentiation, Dukes' stage or site within the colon.

### *Dual staining of tissues for bcl-2 and p53 immunoreactivity*

Recent work has suggested that wild-type p53 oncoprotein down-regulates the expression of bcl-2 (Miyashita *et al.*, 1994*a*, *b*). A total of 5/19 (26.3%) of adenomas and 12/53 (22.6%) of adenocarcinomas contained areas of both bcl-2 and p53 immunoreactivity. This suggested that these samples contained cells which express both bcl-2 and p53. To investigate this possibility we carried out dual staining for both bcl-2 and p53. In the majority of specimens which were immunopositive for both bcl-2 and p53, the areas of immunoreactivity for these two proteins were topographically distinct. Only in 1/19 adenomas and 2/53 carcinomas did identical cells express both bcl-2 and p53 (Figure 6c). This was an uncommon finding with the great majority of epithelial cells within these three neoplasms expressing either p53 or bcl-2 alone (Figure 6). Even in areas in which the cells were immunopositive for both bcl-2 and p53 there was evidence of reciprocity of their expression. As shown in Figure 6, a few cells stained positive for both bcl-2 and p53. However, the cells with the most intense bcl-2 immunoreactivity stained either weakly or were entirely negative for p53 and vice versa (see cells indicated by small arrows in Figure 6).

### Discussion

In this study we find a lower proportion of carcinomas (36.5%) than adenomas (63.2%) express bcl-2 protein. Similar results have been reported (Öfner *et al.*, 1995;

**Figure 5** Peroxidase staining of p53 protein of the same neoplasms as in Figure 2. Two well-differentiated adenocarcinomas, Dukes' stage B (**a**) and Dukes' stage A (**c**), and a tubullovillous adenoma (**b**). In **a** the same area as in Figure 2a is shown in which the nuclei stain positive for p53. Focal (**b**) and scattered (**c**) patterns of p53 staining are also illustrated (×370).

Sinicrope *et al.*, 1995) though the reduction in bcl-2 expression in carcinomas compared with adenomas is less dramatic than in our series. Two other studies (Bronner *et al.*, 1995; Hague *et al.*, 1994) report that more than 90% of adenomas express bcl-2 and they find no reduction in the proportion of carcinomas expressing bcl-2. The reasons for these discrepancies are unclear though they may be related to methodological differences or to the small sample sizes of these studies. However, the lower rate of bcl-2-positive carcinomas in our series in unlikely to be due to false-negative reporting since there was intense bcl-2 immunoreactivity in lymphocytes in all specimens studied (Figure 2b, insert).

The immunostaining was always confined to the cytoplasm and nuclear membrane as has been previously reported (Merritt *et al.*, 1995; Sinicrope *et al.*, 1995). A striking feature was the focal nature of bcl-2 immunoreactivity in the majority of tumours (84.2%) studied. This is unlikely to be due to lack of reproducibility of bcl-2 immunoreactivity within epithelial cells as the staining of lymphocytes was constant within sections. Nor is it likely that the heterogeneity of bcl-2 expression within the cytoplasm can be explained by differences in the position of cells within the cell cycle (Lu *et al.*, 1994). Differences in the local cellular environment may explain the heterogeneity of bcl-2 expression such as variation of growth factor concentration or lymphocytic infiltration (Öfner *et al.*, 1995). However, little is known about extracellular signals capable of regulating bcl-2 expression.

The cause of bcl-2 expression in adenomas and in morphologically normal crypts adjacent to cancers is unclear. The simplest explanation is that the clone of cells which has developed into the adenoma is derived from a cell at the base of the crypt and the bcl-2 expression which is normal for these crypt base stem cells has been retained. However, there is no evidence to indicate which cells along the crypt/villus axis actually develop into malignant clones, though experiments in mice carrying a truncated *Apc* gene suggest they may arise from the lower proliferative zone of the crypt (Oshima *et al.*, 1995). Other possible explanations include translocation of the *bcl*-2 gene to another chromosomal site such as the t(14:18) translocation in non-Hodgkin's B-cell lymphomas; this places *bcl*-2 in close proximity to powerful enhancer elements in the Ig heavy chain locus (Korsmeyer, 1992). Alternatively, mutation of the *bcl*-2 promoter causing deregulated protein expression or mutation of the bcl-2 protein itself, thereby increasing its half-life, are other possible mechanisms. However, there is no information on the incidence of *bcl*-2 translocations or mutations in human colorectal cancer, though a single mutation of uncertain physiological significance has been detected in a human colorectal cancer cell line (Pietenpol *et al.*, 1994). Another mechanism is that loss of wild-type, functional p53 could lead to deregulated expression of bcl-2 protein. The *bcl*-2 gene is transcriptionally repressed by p53 and loss of p53 is sufficient to up-regulate bcl-2 (Miyashita, 1994*a* and *b*). However, such a mechanism is unlikely to account for the expression of bcl-2 in many of the adenomas since mutation and loss of heterozygosity of p53 occurs typically at the transition between adenoma and carcinoma (Baker *et al.*, 1990). Our observation of the high rate of bcl-2 positive adenomas and its more extensive expression in histologically normal crypts in regions adjacent to adenomas suggests that bcl-2 expression is an early event in adenoma formation and occurs before changes in p53. Interestingly, we

**Figure 6** AEC staining for Bcl-2 protein (red) and 3,3'diaminobenzidine + nickel chloride staining of p53 protein (blue-black) of a rectal tubullovillous adenoma. Cells that stain positive for Bcl-2 stain weakly or are entirely negative for p53 (small arrows). Occasional cells that stain for both Bcl-2 and p53 are shown (**c**, large-headed arrow). **a** and **b**, ×275; **c**, ×500.

observed that bcl-2 immunoreactivity was more intense adjacent to Peyer's patches. This raises the possibility that epigenetic factors such as secreted cellular products may influence bcl-2 expression. Alternatively, changes in extracellular matrix, that we have recently shown to regulate bcl-2 expression, may be important (Dive *et al.*, 1995).

There are a number of reasons why bcl-2 expression may be lost during the evolution of colorectal cancer. In the present study we demonstrate a clear inverse relationship between bcl-2 and p53 expression in both adenomas and carcinomas, confirming our previous preliminary observations (Merritt *et al.*, 1995). Although 26% of adenomas and 20% of cancers contained areas of bcl-2 and p53 immunoreactivity, double staining demonstrated that only 5% of the adenomas and 4% of the carcinomas contained cells which actually expressed both proteins. Even in the uncommon instances where cells did express both proteins there was evidence of reciprocity of bcl-2 and p53 expression in the majority of cells (Figure 6). These results are in accordance with previous data demonstrating that wild-type p53 (Selvakumaran *et al.*, 1994) and some p53 mutants (mut 175) (Haldar *et al.*, 1994) down-regulate *bcl*-2 by binding to a transcriptional silencer element within the *bcl*-2 promoter (Miyashita *et al.*, 1994*a*). Although the p53 antibody used in this study could detect both stabilised wild-type and most p53 mutants, it is likely that the majority, but not necessarily all, of the p53 immunoreactivity reflected mutant rather than wild-type p53 (Baker *et al.*, 1990; Hall and Lane, 1994). This suggests that either most p53 mutants can transcriptionally repress *bcl*-2, which is unlikely, or other mechanisms account for the loss of bcl-2 expression. For example, there might be loss of heterozygosity of the *bcl*-2 gene, together with mutation and inactivation of the other allele. The *bcl*-2 gene locus is on chromosome segment 18q21.3 (Tsujimoto *et al.*, 1985). Loss of chromosome 18q occurs in 69% of colorectal cancer (Jen *et al.*, 1994) and allelic loss of the *bcl*-2 gene locus has been observed in 60% of colorectal cancers (Ayhan *et al.*, 1994). Alternatively, high levels of bcl-2 may not be required to prevent apoptosis when tumours acquire p53 mutations and other survival factors come into play.

Both p53 and bcl-2 regulate radiation-induced apoptosis in colorectal epithelium (Merritt *et al.*, 1994, 1995). Studies on mouse lymphocytes indicate that bcl-2 is able to suppress the p53-mediated apoptosis induced by DNA damage (Marin *et al.*, 1994). This raises the possibility that knowledge of bcl-2 status might provide information predicting the response of colorectal tumours to radio- and chemotherapy and patient survival. Recent results suggest that bcl-2 expression is an independent prognostic factor associated with favourable clinical outcome (Öfner *et al.*, 1995), suggesting that loss of bcl-2 expression is associated with either the development of other more potent survival factors or alternatively loss of pro-apoptotic genes such as *bax* (Oltvai *et al.*, 1993) or *bak* (Farrow *et al.*, 1995).

In summary, we have demonstrated that bcl-2 is expressed in a high proportion of adenomas but is often lost during progression to carcinoma and we have shown an inverse relationship between bcl-2 and p53 expression in cells of both colorectal adenomas and carcinomas. Further studies of other regulators of apoptosis are required before either the ability of colorectal tumours to undergo apoptosis can be predicted or the value of p53 or bcl-2 as prognostic indicators is established.

**Acknowledgements**
The authors thank Dr Gordon Armstrong for his help with histological interpretation. This work was supported by the Cancer Research Campaign including (CRC) grant (SP 2234) to JAH and a grant from the British Digestive Foundation to AJMW and grants from CNR ACRO 94.0177.39 to AB and MB.

### References

AYHAN A, YASUI W, YOKOZAKI H, SETO M, UEDA R AND TAHARA E. (1994). Loss of heterozygosity at the bcl-2 gene locus and expression of bcl-2 in human gastric and colorectal carcinomas. *Jpn. J. Cancer Res.*, **85**, 584–591.

BAKER SJ, PREISINGER AC, JESSUP JM, PARASKEVA C, MARKOWITZ S, WILLSON JKV, HAMILTON S AND VOGELSTEIN B. (1990). p53 gene mutations occur in combination with p17 allelic deletions as late events in colorectal tumorigenesis. *Cancer Res.*, **50**, 7717–7722.

BRONNER MP, CULIN C, REED JC AND FURTH EE. (1995). The bcl-2 proto-oncogene and the gastrointestinal epithelial tumor progression model. *Am. J. Pathol.*, **146**, 20–26.

DIVE C, PULLAN S, WILSON J, HICKMAN J, TILLY J AND STREULI C. (1995). Requirement of basement membrane for the suppression of apoptosis in mammary epithelium. *Proc. Am. Assoc. Cancer*, **36**, 1.

FARROW SN, WHITE JHM, MARTINOU I, RAVEN T, PUN K, GRINHAM CJ, MARTINOU J AND BROWN R. (1995). Cloning of a bcl-2 homologue by interaction with adenovirus E1B 19K. *Nature*, **374**, 731–733.

FISHER TC, MILNER AE, GREGORY CD, JACKMAN AL, AHERNE GW, HARTLEY JA, DIVE C AND HICKMAN JA. (1993). bcl-2 modulation of apoptosis induced by anticancer drugs: resistance to thymidylate stress is independent of classical resistance pathways. *Cancer Res.*, **53**, 3321–3326.

FISHER CJ, GILLETT CE, VOJTESEK B, BARNES DM AND MILLIS RR. (1994). Problems with p53 immunohistochemical staining: the effect of fixation and variation in the methods of evaluation. *Br. J. Cancer*, **69**, 26–31.

HAGUE A, MOORGHEN M, HICKS D, CHAPMAN M AND PARASKEVA C. (1994). BCL-2 expression in human colorectal adenomas and carcinomas. *Oncogene*, **9**, 3367–3370.

HALDAR S, NEGRINI M, MONNE M, SABBIONI S AND CROCE CM. (1994). Down-regulation of bcl-2 and p53 in breast cancer cells. *Cancer Res.*, **54**, 2095–2097.

HALL PA AND LANE DP. (1994). p53 in tumour pathology: can we trust immunohistochemistry? - revisited! *J. Pathol.*, **172**, 1–4.

HOCKENBURY DM, ZUTTER M, HICKHEY B, NAHM M AND KORSMEYER SJ. (1991). Bcl-2 protein is topographically restricted in tissues characterised by apoptotic cell death. *Proc. Natl Acad. Sci. USA*, **88**, 6961–6965.

JASS JR, ATKIN WS, CUZICK J, BUSSEY HJ, MORSON BC, NORTHOVER JM AND TODD IP. (1986). The grading of rectal cancer: historical perspectives and a multivariate analysis of 447 cases. *Histopathology*, **10**, 437–459.

JEN J, KIM H, PIANTADOSI S, LIU Z-F, LEVITT RC, SISTONEN P, KINZLER KW, VOGELSTEIN B AND HAMILTON SR. (1994). Allelic loss of chromosome 18q and the prognosis in colorectal cancer. *N. Engl. J. Med.*, **331**, 213–221.

KORSMEYER SJ. (1992). Bcl-2 initiates a new category of oncogenes: regulators of cell death. *Blood*, **80**, 879–886.

LU QL, HANBY AM, NASSER-HAJIBAGHERI MA, GSCHMEISSNER SE, LU PJ, TAYLOR-PAPADIMITRIOU J, KRAJEWSKI S, REED JC AND WRIGHT NA. (1994). Bcl-2 protein localises to the chromosomes of mitotic nuclei and is correlated with the cell cycle in cultured epithelial cell lines. *J. Cell. Sci.*, **107**, 363–371.

MARIN MC, HSU B, MEYN RE, DONEHOWER LA, EL-NAGGAR AK AND MCDONNELL TJ. (1994). Evidence that p53 and bcl-2 are regulators of a common cell death pathway important for *in vivo* lymphomagenesis. *Oncogene*, **9**, 3107–3112.

MERRITT AJ, POTTEN CS, KEMP CJ, HICKMAN JA, BALMAIN A, LANE DP AND HALL PA. (1994). The role of p53 in spontaneous and radiation-induced apoptosis in the gastrointestinal tract of normal and p53-deficient mice. *Cancer Res.*, **54**, 614–617.

MERRITT AJ, POTTEN CS, WATSON AJM, LOH DY, NAKAYAMA K, NAKAYAMA K AND HICKMAN JA. (1995). Differential expression of bcl-2 in intestinal epithelia. Correlation with attenuation of apoptosis in colonic crypts and the incidence of colonic neoplasia. *J. Cell. Sci.*, **108**, 2261–2271.

MIYASHITA T, HARIGAI M, HANADA M AND REED JC. (1994*a*). Identification of a p53-dependent negative response element in the bcl-2 gene. *Cancer Res.*, **54**, 3131–3135.

MIYASHITA T, KRAJEWSKI S, KRAJEWSKI M, WANG HG, LIN HK, LIEBERMANN DA, HOFFMAN B AND REED JC. (1994*b*). Tumour suppressor p53 is a regulator of bcl-2 and bax gene expression in vitro and in vivo. *Oncogene*, **9**, 1799–1805.

ÖFNER D, REIHEMANN K, MAIER H, RIEDMANN B, NEHODA H, TÖTSCH M, BÖCKER W, JASANI B AND SCHMID KW. (1995). Immunohistochemically detectable bcl-2 expression in colorectal carcinoma: correlation with tumour stage and patient survival. *Br. J. Cancer*, **72**, 981–985.

OLTVAI ZN, MILLIMAN CL AND KORSMEYER SJ. (1993). Bcl-2 heterodimerizes in vivo with a conserved homolog, bax, that accelerates programmed cell death. *Cell*, **74**, 609–619.

OSHIMA M, OSHIMA H, KITAGAWA K, KOBAYASHI M, ITAKURA C AND TAKETO M. (1995). Loss of *Apc* heterozygosity and abnormal tissue building in nascent intestinal polyps in mice carrying a truncated *Apc* gene. *Proc. Natl Acad. Sci.*, **92**, 4482–4486.

PIETENPOL JA, PAPADOPOULOS N, MARKOWITZ S, WILLSON JKV, KINZLER KW AND VOGELSTEIN B. (1994). Paradoxical inhibition of solid tumour cell growth by bcl-2. *Cancer Res.*, **54**, 3714–3717.

POTTEN CS. (1992). The significance of spontaneous and induced apoptosis in the gastrointestinal tract. *Cancer Met. Rev.*, **11**, 179–195.

POTTEN CS, LI YQ, O'CONNOR PJ AND WINTON DJ. (1992). A possible explanation for the differential cancer incidence in the intestine, based on the distribution of cytotoxic effects of carcinogens in the murine large bowel. *Carcinogenesis*, **13**, 2305–2312.

SELVAKUMARAN M, LIN HK, MIYASHITA T, WANG HG, KRAJEWSKI S, REED JC, HOFFMAN B AND LIEBERMANN D. (1994). Immediate early up-regulation of bax expression by p53 but not TGF-beta 1: a paradigm for distinct apoptotic pathways. *Oncogene*, **9**, 1791–1798.

SINICROPE FA, RUAN SB, CLEARY KR, STEPHENS LC, LEE JJ AND LEVIN B. (1995). bcl-2 and p53 oncoprotein expression during colorectal tumorigenesis. *Cancer Res.*, **55**, 237–241.

TSUJIMOTO Y, GORHAM J, COSSMAN J, JAFFE E AND CROCE CM. (1985). The t(14:18) chromosome translocations involved in B-cell neoplasms result from mistakes in VDJ joining. *Science*, **229**, 1390–1393.

WATSON AJM. (1995). Manipulation of cell death – the development of novel strategies for the treatment of gastrointestinal disease. *Aliment. Pharmacol. Ther.*, **9**, 215–226.